# Comparison of the Clinical Course of Viral Respiratory Infections in Hospitalized Patients During the 2025/2026 Season in Poland

**DOI:** 10.3390/vaccines14080651

**Published:** 2026-07-24

**Authors:** Piotr Rzymski, Małgorzata Wajdowicz, Szymon Piaszczyński, Piotr Czupryna, Karolina Turzańska, Monika Pazgan-Simon, Paweł Skwara, Justyna Hlebowicz, Maciej Piaseck, Dorota Zarębska-Michaluk, Katarzyna Sikorska, Robert Flisiak

**Affiliations:** 1 Department of Environmental Medicine, Poznan University of Medical Sciences, 60-806 Poznań, Poland; 2 Medical Center in Łańcut, Clinical Department of Infectious Diseases and Hepatologic Unit, University of Rzeszów, 35-959 Rzeszów, Poland; mwajdowicz@gmail.com; 3 Clinical Department of Infectious Diseases and Hepatology, Medical University of Lodz, 90-419 Łódź, Poland; szpiaszczynski@gmail.com; 4 Department of Infectious Diseases and Neuroinfection, Medical University of Bialystok, 15-540 Białystok, Poland; piotr.czupryna@umb.edu.pl; 5 Department of Infectious Diseases and Hepatology, Faculty of Medicine, Collegium Medicum Bydgoszcz, Nicolaus Copernicus University, 87-100 Toruń, Poland; karolina.turzanska@cm.umk.pl; 6 Department of Infectious Diseases and Hepatology, Wrocław Medical University, 50-370 Wrocław, Poland; monika.pazgan.simon@gmail.com; 7 Department of Infectious and Tropical Diseases, Jagiellonian University, 30-688 Kraków, Poland; pawel.skwara@uj.edu.pl; 8 Department of Infectious Diseases and Hepatology, Medical University of Bialystok, 15-540 Białystok, Poland; justyna.hlebowicz@umb.edu.pl (J.H.); robert.flisiak1@gmail.com (R.F.); 9 Department of Infectious Diseases and Hepatology, Medical University of Silesia, 41-500 Katowice, Poland; maciejp67@gmail.com; 10 Department of Infectious Diseases and Allergology, Jan Kochanowski University, 25-317 Kielce, Poland; dorota1010@tlen.pl; 11 Division of Tropical and Parasitic Diseases, Institute of Maritime and Tropical Medicine, Medical University of Gdansk, 80-210 Gdynia, Poland; katarzyna.sikorska@gumed.edu.pl

**Keywords:** respiratory infections, respiratory syncytial virus, SARS-CoV-2, COVID-19, influenza, epidemiology, clinical severity, infectious diseases, public health

## Abstract

**Background/Objectives:** SARS-CoV-2, influenza viruses, and respiratory syncytial virus (RSV) remain major causes of adult hospitalizations, but contemporary comparative data are limited. This study compared the epidemiological characteristics, clinical presentation, and outcomes of adults hospitalized with these infections during the 2025/2026 epidemic season in Poland. **Methods:** We conducted a retrospective multicenter study of consecutive adults hospitalized with laboratory-confirmed COVID-19, influenza, or RSV infection between September 2025 and April 2026. Demographic characteristics, comorbidities, vaccination status, clinical features, laboratory findings, and outcomes were analyzed. Independent predictors of in-hospital mortality were identified using multivariable logistic regression. **Results:** The study included 604 patients: 255 with COVID-19, 314 with influenza, and 35 with RSV infection. Distinct seasonal patterns were observed, with COVID-19 peaking in autumn, influenza in winter, and RSV in early spring. Most hospitalized patients were elderly and unvaccinated. RSV patients were older, more frequently affected by multimorbidity, ischemic heart disease, and cancer, and showed the greatest respiratory impairment, including the highest rates of hypoxemia and pneumonia. Influenza was characterized by more frequent fever, headache, and myalgia. Despite lower pneumonia rates, COVID-19 was associated with the highest in-hospital mortality (13.7%) and remained an independent predictor of death (aOR = 3.23, 95%CI: 1.68–6.22). Antibiotic use was common across all cohorts (62–77%). **Conclusions:** COVID-19 remained associated with the highest mortality among hospitalized adults, whereas RSV contributed substantially to respiratory morbidity in older individuals. These findings support improved vaccination uptake, continued surveillance, hospital preparedness, and antimicrobial stewardship.

## 1. Introduction

Viral respiratory infections are among the leading causes of morbidity and mortality worldwide, presenting a continuous and substantial challenge to healthcare systems globally [1,2,3]. While historically often associated with pediatric or geriatric populations, contemporary epidemiological data underscore that these pathogens may exert a profound socioeconomic and clinical toll across the entire adult population [4,5,6]. Among the vast array of respiratory viruses, severe acute respiratory syndrome coronavirus 2 (SARS-CoV-2), influenza viruses, and respiratory syncytial virus (RSV) have emerged as the most burdensome pathogens capable of causing severe lower respiratory tract infections, exacerbating underlying chronic conditions, increasing the risk of secondary bacterial invasions, and driving adult hospitalizations [7,8,9].

These three viral pathogens exhibit distinct yet frequently overlapping seasonal patterns in the temperate climate zone, typically concentrating their impact from late summer through early spring [10,11]. Managing this seasonal surge has been historically complicated by diagnostic limitations; however, the clinical landscape has shifted significantly. While resource-intensive molecular methods like real-time polymerase chain reaction (RT-PCR) remain the gold standard, they are not always readily available or accessible in acute clinical settings [12,13]. Today, the widespread availability of rapid antigen tests, frequently in the form of multiplex assays, allows for timely, inexpensive point-of-care differential diagnosis, optimizing patient isolation and targeted treatment strategies [14,15]. Furthermore, a critical commonality among SARS-CoV-2, influenza, and RSV is that all three are now vaccine-preventable in adults. The deployment of effective vaccines offers a powerful mechanism to mitigate severe outcomes, yet vaccine uptake and effectiveness remain variable [16].

Because the seasonal burdens of COVID-19, influenza, and RSV can overlap or sequentially extend over a prolonged period [17], understanding their concurrent dynamics is pivotal. This evaluation must occur on multiple tiers, including population-wise epidemiological tracking and hospital-wise capacity management. Compounding this challenge is the intrinsic nature of these pathogens as all three are RNA viruses [18]. Consequently, they are inherently prone to genetic mutations, though their mutation rates and evolutionary mechanisms vary significantly, ranging from the rapid antigenic drift and shift of influenza viruses [19] and the volatile evolution of SARS-CoV-2 driven by variant-specific population immunity [20], to the more stable yet clinically relevant mutations of RSV [21,22]. These evolutionary dynamics, combined with shifting population immunity, varying vaccine uptake, and changing healthcare resource capacities, indicate that patient profiles, clinical presentations, and institutional burdens can potentially fluctuate from one epidemic season to the next [23,24,25].

To address critical data gaps regarding contemporary viral co-circulation, the primary outcome of this study was a comprehensive analysis of adult hospitalizations in Poland during the 2025/2026 epidemic season due to COVID-19, influenza, and RSV in real-world settings. These three viruses were selected because they represent major vaccine-preventable causes of adult respiratory infection-related hospitalizations, are routinely considered in differential diagnosis during the respiratory season, and have direct relevance for adult immunization policy and hospital preparedness. The secondary outcome was to compare patient demographic profiles, underlying comorbidities, clinical markers, and indicators of disease severity. Ultimately, these data seek to inform local clinical guidelines, optimize hospital resource allocation, and strengthen public health immunization strategies in Poland.

## 2. Materials and Methods

### 2.1. Sample Collection

Data for this retrospective study were gathered from adult patients hospitalized between September 2025 and April 2026 across participating medical units involved in the SARSTer and FluTer projects, initiatives conducted under the auspices of the Polish Association of Epidemiologists and Infectiologists. The study population comprised all consecutive adult patients admitted to the leading infectious diseases wards in Poland (Białystok, Bydgoszcz, Gdynia, Katowice, Kielce, Kraków, Łańcut, Łódź, Wrocław) due to acute, laboratory-confirmed infections (with RT-PCR and/or rapid antigen test) caused by SARS-CoV-2, influenza viruses, or respiratory syncytial virus RSV. Patients admitted to the hospital with symptoms of acute respiratory infection were routinely tested with nasal and/or throat samples using RT-PCR or rapid antigen testing. Only patients with symptoms of acute respiratory infection and a positive test result were included in the study. Patients in all participating units were admitted, diagnosed, and treated according to the available recommendations in Poland [26,27,28]. To obtain a real-world cohort of hospitalized adults with acute laboratory-confirmed respiratory viral infections, no exclusion criteria were applied to consecutive eligible admissions. Conversely, patients hospitalized for non-infectious conditions or chronic illnesses lacking an underlying acute viral infection were excluded, as such individuals do not routinely populate these specialized units and fell outside the scope of this investigation.

For all included patients, comprehensive clinical data were extracted from medical records. These parameters encompassed demographic characteristics (age and sex), the presence of pre-existing comorbidities, symptom profile, and several clinical indicators at admission (oxygen saturation levels, serum C-reactive protein (CRP), d-dimer, and counts of lymphocytes and neutrophils). Vaccination status was defined separately for each infection according to receipt of the most recent recommended vaccine against the respective disease before hospitalization. Patients who had not received the most recent recommended vaccine, or for whom such vaccination was not documented, were classified as unvaccinated for that infection. Additionally, clinical outcomes and complications, such as the duration of hospitalization, the occurrence of pneumonia, the use of antibiotics during the course of treatment (though microbiological confirmation of bacterial coinfection was not systematically available), oxygen therapy, and mechanical ventilation, as well as the incidence of death, were also recorded as indicators of the course of the disease.

The study was approved by the Bioethics Committee of Jan Kochanowski University in Kielce (resolution no. 16/2025, approved on 19 March 2025). It has been performed in accordance with the ethical standards laid down in the 1964 Declaration of Helsinki and its subsequent amendments. Given the retrospective design of the research and the strict maintenance of patient anonymity, the requirement for written informed consent was waived.

### 2.2. Statistical Analyses

Statistical analyses were performed to compare the demographic, clinical, and outcome profiles among the COVID-19, influenza, and RSV groups. Continuous variables are presented as mean ± standard deviation for descriptive purposes. Because their distributions deviated from normality (tested with Shapiro–Wilk’s test), between-group comparisons were performed using the Kruskal–Wallis test followed by Dunn’s post hoc test. Because of the limited number of outcome events and the relatively small RSV subgroup, center-adjusted sensitivity analyses were not performed, as such models would be at risk of instability and overfitting. To identify independent predictors of in-hospital mortality, univariate analyses were first performed to compare survivors and deceased individuals. Variables that differed significantly between the two groups in univariate analyses were considered candidate predictors and entered into the multivariable logistic regression model. The dependent variable was in-hospital mortality, coded as 0 for survival and 1 for death. All candidate predictors included in the model were dichotomous variables coded as 0 for absence and 1 for presence of a given characteristic; therefore, assessment of linearity in the logit for continuous variables was not applicable. Multicollinearity among predictors was assessed using variance inflation factors. All values were low, indicating no evidence of problematic multicollinearity among variables included in the model. No missing data were present for the variables included in the model. The results of the multivariable logistic regression were presented as adjusted odds ratio (aOR) with 95% confidence interval and corresponding *p*-value. For all tests, a *p*-value below 0.05 was considered statistically significant.

## 3. Results

### 3.1. Hospitalization Dynamics

**Figure 1:**
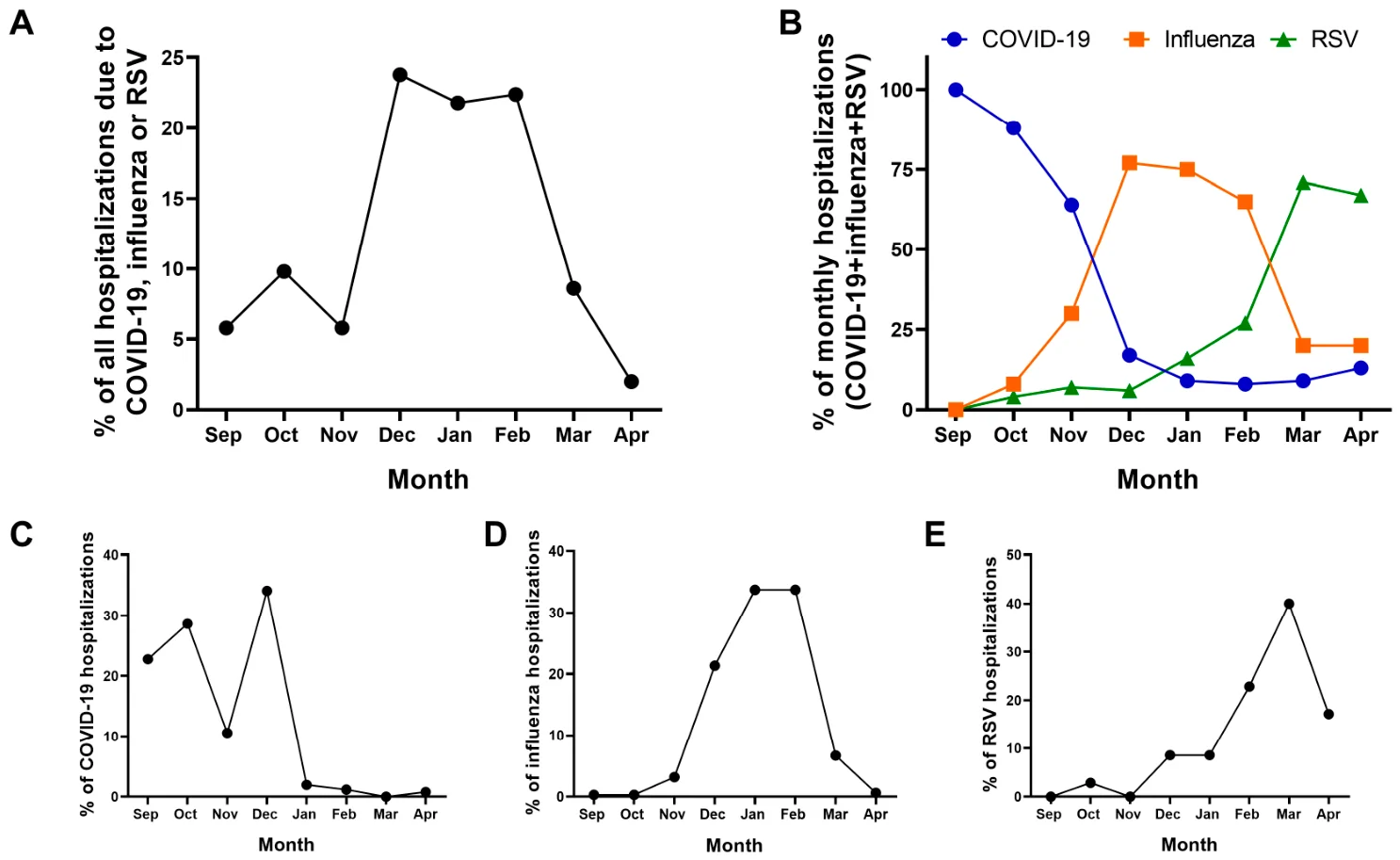
Monthly patterns of hospitalizations due to laboratory-confirmed COVID-19, influenza, and RSV in the 2025/2026 epidemic season. (**A**) Percentage of all included hospitalizations due to COVID-19, influenza, or RSV occurring in each month. (**B**) Relative contribution of COVID-19, influenza, and RSV to all respiratory viral hospitalizations within each month. (**C**–**E**) Monthly distribution of hospitalizations within each infection group: (**C**) COVID-19, (**D**) influenza, and (**E**) RSV.

Overall, 604 patients were hospitalized in the participating infectious disease wards between September 2025 and April 2026, including 255 individuals with COVID-19 (42.2%), 314 with influenza (52.0%), and 35 with RSV (5.8%). No coinfections were recorded in this cohort. December–February was the period most heavily burdened by the respiratory viral infections considered, accounting for 64.3% of all hospitalizations due to COVID-19, influenza, and RSV (Figure 1A). The relative contribution of individual infections varied substantially across the season: COVID-19 predominated in September–November, influenza became the dominant cause of hospitalization from December to February, and RSV accounted for the largest relative share in March and April (Figure 1B). When each infection was analyzed separately across the season, COVID-19 hospitalizations were concentrated mainly in September, October, and December, with the highest proportion observed in December (Figure 1C). Influenza hospitalizations increased from November onward and peaked in January–February 2026 (Figure 1D), whereas RSV hospitalizations increased from December onward and peaked in March 2026 (Figure 1E).

### 3.2. General Patients Profile

Demographic characteristics of the analyzed cohorts are summarized in Table 1. The majority of patients were elderly, although the RSV cohort was significantly older. The vast majority of hospitalized individuals were not vaccinated against the respective disease. No significant differences were observed regarding sex distribution, BMI, or the presence of at least one comorbidity. However, patients hospitalized with RSV had the highest prevalence of multimorbidity (defined as at least two comorbidities), ischemic heart disease, and cancer. Among patients with a specified neoplasm type, prostate cancer, lymphomas, and meningiomas/CNS tumors were the most frequently reported diagnoses, with three cases each. Breast cancer or breast tumor was reported in two patients. Single cases included brain glioma, Kaposi sarcoma, retroperitoneal tumor, ovarian cancer, bladder cancer, and liver cancer. The rates of peripheral vascular disease, arterial hypertension, diabetes, asthma, and chronic obstructive pulmonary disease did not differ significantly among the three cohorts. Immunodeficiency differed significantly among the groups, being most common in patients with COVID-19, followed by RSV and influenza (Table 1). The vast majority of patients hospitalized with influenza were infected with type A viruses (95.2%).

### 3.3. Clinical Course

**Figure 2:**
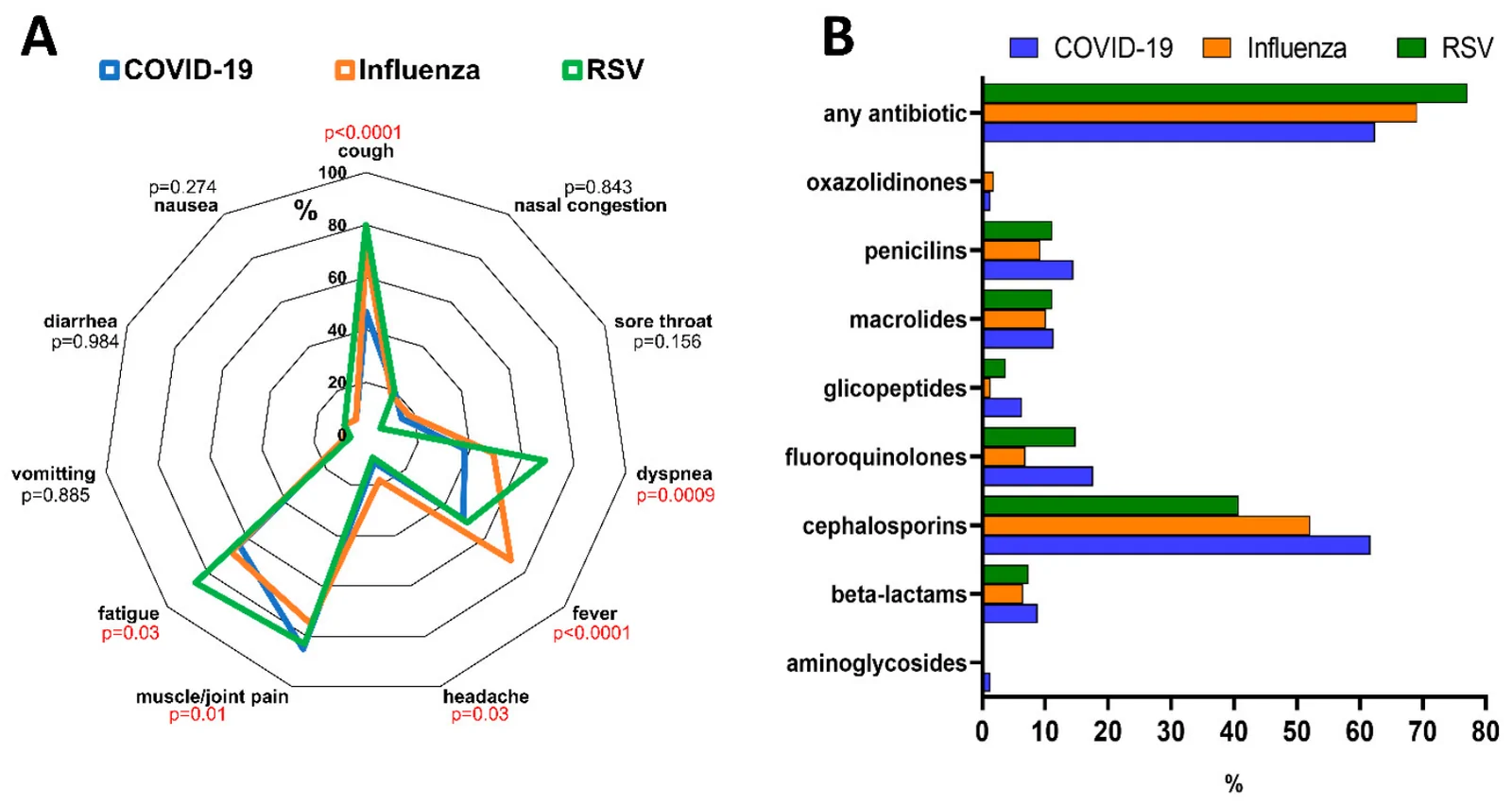
(**A**) Radar plot representing symptoms’ frequency (%) in patients hospitalized with COVID-19, influenza, and RSV in the 2025/2026 epidemic season. (**B**) Utilization of antibiotic classes in three cohorts of hospitalized patients.

Significant differences between the three cohorts were also observed in symptomatology at admission (Figure 2A). Cough was least frequent in COVID-19 (<50% of patients) and was present in the majority of patients hospitalized with influenza (71%) or RSV infection (80%). Fever, muscle/joint pain, and headache were most prevalent in influenza (73%, 25%, and 18% of patients, respectively). Dyspnea was most frequent in RSV (69%), followed by influenza (49%) and COVID-19 (38%). Fatigue was more common in RSV patients (86%) than in those with influenza (68%) or COVID-19 (64%). No differences in the frequency of other symptoms, including nasal congestion, sore throat, nausea, vomiting, and diarrhea, were observed between the COVID-19, influenza, and RSV cohorts.

Some differences in laboratory parameters were also noted between the studied groups (Table 2). Patients hospitalized due to RSV infection had the lowest oxygen saturation, with over half of them having it below 90%. On the other hand, these patients, along with those with influenza, had lower CRP levels than the COVID-19 cohort, though the proportion of individuals with CRP > 100 mg/L did not differ between the groups (Table 2).

### 3.4. Clinical Outcomes

Across the three viral cohorts tracked during the 2025/2026 epidemic season, significant variations were observed in hospitalization length, secondary complications, and survival outcomes (Table 3). The average length of stay spanned 8–9 days across all groups, with no significant difference between cohorts. The highest prevalence of pneumonia was documented among patients with RSV, followed by influenza, and was lowest in the COVID-19 cohort (*p* = 0.04). Although RSV patients also required antibiotic intervention most frequently, the differences in antibiotic administration rates across the three groups did not reach statistical significance (*p* = 0.09). In all three cohorts, the cephalosporins were the most widely used antibiotic classes (Figure 2B). Notably, despite having the shortest average hospital stays and the lowest incidence of pneumonia, patients hospitalized with COVID-19 exhibited a profoundly higher mortality rate compared to those in the influenza and RSV cohorts (*p* = 0.03; Table 3).

Table 4 summarizes the main characteristics of patients with fatal outcomes. There was only one individual (male, aged 52, not vaccinated) with RSV infection who died. The vast majority of patients who died from either COVID-19 or influenza were elderly individuals. Although both groups exhibited high rates of underlying comorbidities, deceased COVID-19 patients were characterized by significantly higher prevalence of multimorbidity, active malignancy, and immunocompromised status compared to the influenza group. In contrast, ischemic heart disease, arterial hypertension, and chronic kidney disease were common and relatively evenly distributed between both cohorts. Vaccination rates prior to admission were notably low among all deceased patients. Upon hospitalization, both groups presented with severe clinical manifestations, with the majority experiencing hypoxemia, developing pneumonia, and requiring antibiotic treatment prior to death (Table 4). In the multivariate logistic model, COVID-19 was an independent predictor of death in the studied cohort, following adjustment for age, sex, comorbidities, immunodeficiency, hypoxia at admission, and pneumonia onset (aOR = 3.23, 95%CI: 1.68–6.22; *p* = 0.0005).

## 4. Discussion

To the best of our knowledge, this is the first study to compare adult hospitalizations in Poland due to influenza, COVID-19, and RSV across a single epidemic season. The presented data demonstrate substantial differences in patient profiles, disease severity, and outcomes among these infections, providing valuable insights into their distinct epidemiological and clinical characteristics. Such comparative data are essential for assessing the burden of respiratory infections, optimizing hospital preparedness and resource allocation, and supporting evidence-based prevention strategies, including vaccination policies and targeted protection of vulnerable populations.

The present findings demonstrate a sustained burden of viral respiratory infection-related hospitalizations extending from September 2025 to April 2026. This observation highlights the prolonged pressure exerted on the healthcare system by the three investigated pathogens and underscores the importance of preventive strategies targeting all of them. Seasonal vaccination against COVID-19 and influenza, as well as single-dose RSV vaccination, are currently available for adults in Poland [29]. Importantly, these preventive measures are fully reimbursed for older adults and can be administered not only in primary healthcare settings but also in pharmacies, thereby facilitating access to immunization. Despite this, influenza vaccination coverage among adults in Poland has remained very low for many consecutive seasons, fluctuating at only several percent in the general adult population and reaching merely the low-teen percentages among individuals aged ≥65 years [30]. Uptake of updated COVID-19 vaccination has been even lower, most likely due to limited public trust in these vaccines, a phenomenon that also appears to be present among medical professionals in Poland. In turn, RSV vaccines became broadly available in Poland only in 2025, and by the end of April 2026, over 600,000 doses had been administered. More than 80% of these doses were given to individuals aged ≥65 years, indicating that vaccine uptake was concentrated in the main age-based target group [31]. However, given that this age group includes over 8 million people in Poland, these figures correspond to only approximately 6% coverage among older adults. Collectively, these observations suggest that the burden observed in the present study persists despite the availability of effective preventive tools, supporting the continued need for evidence-based strategies to improve adult respiratory infection prevention. Further studies on potential barriers to adult immunization are required, as the present study was not designed to explore determinants of vaccine acceptance, uptake, or hesitancy.

Importantly, separate analyses for each infection showed that the epidemic season was reflected in a sequential pattern of hospitalizations, beginning with COVID-19, then influenza, and finally RSV. However, one should note that the observed distribution of hospitalizations may not reflect intrinsic viral seasonality alone, but could also have been influenced by changes in community viral circulation, regional outbreak dynamics, healthcare-seeking behavior, admission thresholds, and testing intensity across the respiratory season. Although RSV had the lowest burden among the three investigated infections, both in terms of hospitalizations and mortality, its increasing contribution toward the end of the respiratory infection season is clinically relevant. In this context, RSV may represent a “final wave” of respiratory burden, occurring after several months of continuous immune system challenge following the preceding viral circulation. Such cumulative seasonal pressure may increase susceptibility to clinical complications (e.g., increased cardiovascular risk), particularly among older adults and individuals with comorbidities, thereby further supporting the rationale for RSV-targeted prevention. However, RSV-related findings should be interpreted with particular caution because the RSV cohort was small and substantially smaller than the influenza and COVID-19 cohorts. This imbalance reduced the statistical power of RSV-related comparisons and increased the uncertainty around estimates for this subgroup. Therefore, statistically significant differences involving RSV should be regarded as exploratory and hypothesis-generating rather than definitive.

The present findings also highlight the still-evolving epidemiological behavior of SARS-CoV-2. As a relatively new human pathogen, its seasonality may continue to change substantially and may also be shaped by population behaviors, including vaccine uptake and naturally acquired immunity. In the current study, the peak of COVID-19 hospitalizations occurred in November 2025, as observed during the 2023/2024 season. However, this pattern differed markedly from the 2024/2025 season, during which the highest numbers of cases and hospitalizations were observed already in August, followed by a gradual decline in subsequent months [23]. These observations indicate that COVID-19 remains an infection with difficult-to-predict epidemiological dynamics. Although increases in SARS-CoV-2 transmission may generally be anticipated in late summer or early autumn, the timing and intensity of subsequent waves may vary considerably between seasons. Therefore, historical seasonal patterns should be interpreted cautiously and cannot be directly extrapolated to predict future epidemic dynamics.

Our findings demonstrate that while some group-level differences in clinical and laboratory characteristics were observed among hospitalized patients, considerable overlap remains, underscoring the challenge of bedside differentiation for these respiratory pathogens. Importantly, these symptom patterns should be interpreted as descriptive and should not be regarded as clinically discriminative, as no formal diagnostic performance or predictive analyses were performed. The lower prevalence of cough, dyspnea, and fatigue in the COVID-19 cohort contrasts with the classic RSV presentation, which emerged as potentially most clinically severe at admission, characterized by the highest rates of fatigue, cough, dyspnea, and profound hypoxemia, with over half of patients presenting with oxygen saturation below 90%. Conversely, influenza was characterized by a more pronounced systemic response, leading to the highest frequencies of fever, myalgia, and headache. These patterns align with previous observations in pediatric populations, indicating that fever was more common with influenza than RSV infection, while nasal blockage, dyspnea, severe cough, and numerous abnormal chest sounds are more frequent with RSV than with COVID-19 or influenza [32,33]. However, given the demographic differences between the studied cohorts, these findings should be regarded as exploratory. Moreover, owing to the limited size of the RSV subgroup, they should be interpreted cautiously and not considered conclusive evidence of a distinct RSV phenotype or greater clinical severity at admission.

Interestingly, despite the greater respiratory distress and hypoxemia observed in RSV patients, the COVID-19 cohort exhibited significantly higher baseline CRP levels. This mismatch between severe local respiratory dysfunction in RSV and heightened systemic inflammation in COVID-19 may reflect differences in underlying pathophysiological mechanisms, potentially including the endothelial and systemic inflammatory cascades characteristic of SARS-CoV-2 [34,35]. However, as this was an observational real-world study and these comparisons were not adjusted for age, multimorbidity, immunodeficiency, bacterial coinfection, viral genomic characteristics, or other potential modifiers of inflammatory response, differences in CRP concentrations and oxygen saturation cannot be attributed exclusively to viral etiology and should be interpreted as descriptive findings. Moreover, because symptoms such as nasal congestion, sore throat, and gastrointestinal issues occurred at identical frequencies across all three cohorts, the observed clinical patterns cannot be used to reliably distinguish viral etiology at the individual-patient level. Ultimately, these findings reinforce that while clinical trends can guide initial suspicion, definitive molecular/antigen testing remains a necessity for accurate diagnosis and tailored therapeutic intervention.

Our study also shows that among the three major viral respiratory infections, COVID-19 remains the most severe, with the highest fatality rate (14%) among hospitalized patients and threefold higher odds of death after adjusting for confounding variables (age, sex, comorbidities, immunodeficiency, hypoxia at admission, and pneumonia onset). Previous comparisons of these three infections in adults have yielded mixed results. For example, Surie et al. showed that although RSV was less common during the 2022/2023 season in older adults in the United States, it was often more severe than COVID-19 or influenza in vaccinated individuals, with higher odds of mechanical ventilation or death [36]. In turn, a large US cohort study conducted between 2021 and 2024 found that COVID-19 was associated with a higher risk of in-hospital death than influenza, while RSV and influenza showed similar severity [37]. A nationwide study from Singapore demonstrated that RSV-related hospitalizations were more severe than those caused by influenza, whereas unboosted patients infected with the XBB SARS-CoV-2 subvariant had a higher mortality risk [38]. In our previous work, encompassing the latter part of 2025, COVID-19 was also associated with higher odds of death than influenza, and these findings are confirmed here for the 2025/2026 epidemic season [39]. Even in the 2024/2025 season, influenza, despite an unprecedented burden in Poland, was associated with a lower in-hospital mortality rate (6–11%) [24,40] than COVID-19 in the present work (14%).

Altogether, these data underscore a critical public health paradox defining the current post-pandemic era: while public risk perception of COVID-19 has significantly waned, SARS-CoV-2 remains an epidemiologically volatile and highly transmissible pathogen that continues to inflict a disproportionate burden on both individual health and acute care infrastructure. The relative historical novelty of the virus, compounded by its ongoing mutational variance, introduces a persistent layer of clinical unpredictability. Consequently, robust mitigation strategies remain essential; however, the execution of these interventions faces profound systemic barriers, primarily driven by entrenched vaccine hesitancy, booster fatigue, and a shifting sociopolitical climate that increasingly resists continuous immunization campaigns [41,42].

Another important observation of our study is the substantial use of antibiotics, representing eight different classes in total (with cephalosporins being most frequently utilized), among patients hospitalized due to viral respiratory infections, ranging from 62% in COVID-19 to 77% in RSV. The frequency of antibiotic use in individual diseases coincided with the frequency of diagnosed pneumonia. Therefore, such a high proportion may reflect both clinically suspected or confirmed secondary bacterial infections, most commonly caused by *Streptococcus pneumoniae*, *Haemophilus influenzae*, and *Staphylococcus aureus* [43,44,45], and empirical antibiotic use for presumed bacterial superinfections [46]. However, bacterial coinfections and antimicrobial resistance were not directly assessed in the present study, and, therefore, these findings should be interpreted as indicating substantial antibiotic exposure rather than evidence of inappropriate antibiotic use or antimicrobial resistance. Such a high exposure was also shown previously. For example, in the United Kingdom, an estimated 2.1% of antibiotics were attributable to RSV infections, with the elderly population contributing the greatest volume [47]. In this broader public health context, viral respiratory infections may indirectly contribute to the growing burden of antimicrobial resistance, which is already estimated to be associated with over 4.5 million deaths annually and is projected to exceed 8 million by 2050 [48]. In light of these observations, vaccination may be regarded as an important strategy for reducing antibiotic consumption and, consequently, as a potential contributor to antimicrobial resistance mitigation, a benefit that has been most clearly demonstrated to date for influenza vaccination [49,50].

Our study has several limitations. First, its retrospective hospital-based design limits causal inference and makes the analysis dependent on the completeness and accuracy of routinely collected medical records. Data on time since vaccination and antibody titers were not available, precluding assessment of their relationship with disease severity or outcomes. The study covered a single epidemic season, from September 2025 to April 2026, and may therefore not be generalizable to other seasons, during which viral circulation, influenza subtypes, SARS-CoV-2 variants, population immunity, testing practices, and healthcare utilization may differ. Although the study was multicenter, center-level effects cannot be excluded, as participating hospitals may have differed in patient populations, admission thresholds, diagnostic work-up, testing intensity, treatment strategies, and discharge policies. Such inter-hospital variability, including possible changes in testing intensity over the respiratory season, may have influenced the observed distribution of influenza, COVID-19, and RSV hospitalizations. Potential biases in detection and admission thresholds should also be considered. In routine clinical practice, RSV testing in adults may be more likely to be performed in older patients, those with multimorbidity, or those presenting with more severe respiratory compromise, which could have led to underdetection of milder RSV-related hospitalizations and overrepresentation of more severe RSV cases. This is particularly relevant because rapid antigen tests for RSV are less sensitive in adults than molecular assays; therefore, negative antigen results may not exclude RSV infection with the same reliability as PCR-based testing [22,51]. Although multivariable regression was used to adjust for selected confounders, residual confounding cannot be ruled out, particularly because some potentially relevant clinical variables were unavailable. We also lacked detailed genomic data enabling differentiation of SARS-CoV-2 sublineages, influenza virus subtypes beyond the type A/B distinction, and RSV antigenic groups, all of which may influence clinical severity and outcome [22,23,52,53]. Consequently, we could not assess the match between the viral sublineages or subtypes infecting individual patients and the antigenic composition of the vaccines administered against the respective viruses, nor could we evaluate whether a possible vaccine–virus mismatch may have influenced disease severity or outcomes. Another limitation is that detailed information on prior SARS-CoV-2, influenza, or RSV infections was not available. Therefore, the potential effects of infection-acquired immunity and hybrid immunity could not be accounted for. This is particularly relevant for COVID-19 and influenza, for which previous infection and vaccination history may influence disease severity, clinical course, and outcomes. Consequently, observed differences in severity and mortality between viral groups may have been partly confounded by unmeasured differences in prior immune exposure and should not be interpreted as reflecting viral etiology alone. In addition, although antibiotic use was recorded, microbiological confirmation of bacterial coinfection was not systematically available; therefore, we could not determine whether antibiotic therapy reflected confirmed bacterial infection, clinically suspected superinfection, or potentially unnecessary empirical treatment. Similarly, information on prior outpatient antiviral treatment before hospital admission was not available, which may have influenced disease severity at presentation and subsequent outcomes. Another limitation is the relatively small RSV cohort, comprising only 35 patients and substantially smaller than the influenza and COVID-19 groups. This imbalance limited the statistical power of comparisons involving RSV and increased the uncertainty of RSV-related estimates. As a result, statistically significant differences involving RSV should be interpreted with caution, and non-significant findings should not be taken as evidence of no difference. Further studies are needed to more precisely characterize the clinical profile, severity, and outcomes of RSV-related hospitalizations in adults in Poland.

## 5. Conclusions

The 2025/2026 epidemic season in Poland demonstrated that COVID-19, influenza, and RSV led to a prolonged sequence of adult hospitalizations spanning several months, with COVID-19 predominating earlier in the season, influenza contributing mainly during the winter peak, and RSV appearing later. This temporal pattern, although descriptive and season-specific, suggests that hospital preparedness for viral respiratory infections should not be limited to a single winter peak but should cover the full respiratory season, with flexible allocation of diagnostic capacity, isolation resources, oxygen support, and clinical staffing according to changing viral circulation. The observed differences in patient profiles, clinical presentation, and outcomes further indicate that these infections should be monitored jointly rather than in isolation, while molecular or antigen testing remains essential because clinical features alone are not sufficiently discriminative. The low documented vaccination coverage among hospitalized patients highlights the need to better monitor and address missed opportunities for adult immunization, although this study did not evaluate vaccine effectiveness or determinants of vaccine uptake. Finally, the widespread use of antibiotics across all three viral infections underscores the importance of antimicrobial stewardship and improved assessment of suspected bacterial coinfection in hospitalized patients with viral respiratory disease. Overall, these findings support integrated seasonal surveillance, time-sensitive hospital preparedness, and prevention strategies tailored to the real-world burden of adult COVID-19, influenza, and RSV hospitalizations.

## Figures and Tables

**Table 1:** General characteristics of patients hospitalized with COVID-19, influenza, and RSV in the 2025/2026 epidemic season.

	COVID-19 (*n* = 255)	Influenza (*n* = 314)	RSV (*n* = 35)	*p*-Value
Age, years, mean ± SD (min–max)	73.4 ± 16.9 (19–99) a	69.6 ± 17.6 (18–99) b	75.8 ± 14.7 (36–96) a	**0.001**
Age ≥ 65 years, %, (*n*) *	79.6 (203)	71.7 (225)	85.7 (30)	**0.03**
Male sex, % (*n*)	45.9 (117)	47.3 (148)	51.4 (18)	0.813
BMI, mean ± SD	26.5 ± 5.6	26.8 ± 5.4	26.6 ± 5.6	0.954
Obesity (BMI ≥ 30 kg/m^2^)	14.1 (36)	17.5 (55)	22.9 (8)	0.314
Vaccinated, % (*n*)	13.3 (34)	10.2 (32)	17.1 (6)	0.319
Any comorbidity, % (*n*)	73.3 (187)	72.9 (229)	88.6 (31)	0.128
Multimorbidity **, % (*n*)	51.4 (131)	40.1 (126)	65.7 (23)	**0.002**
Ischemic heart disease, % (*n*)	37.3 (95)	32.8 (103)	54.3 (19)	**0.04**
Peripheral vascular disease, % (*n*)	32.6 (83)	33.8 (106)	51.4 (18)	0.08
Arterial hypertension, % (*n*)	65.1 (166)	66.2 (208)	71.4 (25)	0.756
Diabetes, % (*n*)	30.6 (78)	29.0 (91)	20.0 (7)	0.432
Cancer, % (*n*)	22.0 (56)	12.4 (39)	28.6 (10)	**0.002**
Chronic kidney disease, % (*n*)	16.1 (41)	15.6 (49)	31.4 (11)	0.05
Asthma, % (*n*)	3.1 (8)	5.7 (18)	5.7 (2)	0.326
Chronic obstructive pulmonary disease, % (*n*)	9.4 (24)	12.4 (39)	20.0 (7)	0.149
Immunodeficiency, % (*n*)	16.1 (41)	8.6 (27)	11.4 (4)	**0.02**

Different letters (a, b) in the row denote statistical differences between disease groups in the post hoc test following Kruskal–Wallis ANOVA (*p* < 0.05). *—A ≥ 65 years cutoff was used because this age group is directly relevant to respiratory virus prevention policy in Poland: individuals aged ≥65 years are eligible for fully reimbursed RSV and influenza vaccines, while also remaining at increased risk of severe COVID-19 outcomes. Therefore, reporting the proportion of patients aged ≥65 years helps contextualize the hospitalized cohort in relation to vaccine-preventable high-risk groups. **—defined as the co-occurrence of two or more chronic conditions. *p*-values below 0.05 were bolded.

**Table 2:** Baseline laboratory parameters of patients hospitalized with COVID-19, influenza, and RSV in the 2025/2026 epidemic season.

	COVID-19 (*n* = 255)	Influenza (*n* = 314)	RSV (*n* = 35)	*p*-Value
SpO_2_, %, mean ± SD	92.4 ± 7.1 a	91.8 ± 8.4 ab	90.1 ± 6.0 b	**0.006**
SpO_2_ < 90%, % (*n*)	23.3 (53)	29.7 (88)	54.3 (19)	**0.0006**
CRP, mg/L, mean ± SD	188.0 ± 1943.4 a	77.6 ±69.6 b	88.0 ± 108.8 b	**0.009**
CRP > 100 mg/L, % (*n*)	22.8 (58)	25.8 (81)	22.9 (8)	0.685
D-dimer, ng/mL, mean ± SD	2181.3 ± 6042.6	1985.1 ± 4909.7	2711.0 ± 6635.0	0.577
Lymphocytes, ×10^3^/μL, mean ± SD	2.6 ± 3.5	2.1 ± 3.2	1.8 ± 3.7	0.113
Neutrophils, ×10^3^/μL, mean ± SD	5.5 ± 3.4	5.5 ± 5.2	6.2 ± 4.2	0.125
Oxygen therapy, % (*n*)	47.7 (121)	48.6 (152)	71.4 (25)	**0.03**
Mechanical ventilation, % (*n*)	4.7 (12)	6.1 (19)	2.9 (1)	0.622

Different letters (a, b) in the row denote statistical differences between disease groups in the post hoc Dunn’s test following Kruskal–Wallis ANOVA (*p* < 0.05). CRP—C-reactive protein; SpO_2_—oxygen saturation. *p*-values below 0.05 were bolded.

**Table 3:** Clinical outcomes in patients hospitalized with COVID-19, influenza, and RSV in the 2025/2026 epidemic season.

	COVID-19 (*n* = 255)	Influenza (*n* = 314)	RSV (*n* = 35)	*p*-Value
Hospitalization length, days, mean ± SD	8.2 ± 6.4	9.2 ± 7.2	8.9 ± 4.2	0.05
Pneumonia, % (*n*)	45.5 (116)	54.8 (172)	68.6 (24)	**0.04**
Need for antibiotics, % (*n)*	62.4 (159)	69.1 (217)	77.1 (27)	0.09
Death, % (*n*)	13.7 (35)	8.0 (25)	2.9 (1)	**0.03**

*p*-values below 0.05 were bolded.

**Table 4:** Characteristics of patients hospitalized with COVID-19, influenza, and RSV with fatal outcome.

	COVID-19 (*n* = 35)	Influenza (*n* = 25)	RSV (*n* = 1)	COVID-19 vs. Influenza *p*-Value **
Age > 65 years, %, (*n*)	91.5 (32)	72.0 (18)	0 (0)	**0.04**
Male sex, % (*n*)	54.3 (19)	48.0 (12)	0 (0)	0.631
Obesity (BMI ≥ 30 kg/m^2^)	5.7 (2)	12.0 (3)	100 (1)	0.385
Vaccinated, % (*n*)	14.3 (5)	4.0 (1)	0 (0)	0.190
Any comorbidity, % (*n*)	88.6 (31)	72.0 (18)	100 (1)	0.102
Multimorbidity *, % (*n*)	71.5 (25)	36.0 (9)	100 (1)	**0.006**
Ischemic heart disease, % (*n*)	40.0 (14)	40.0 (10)	0 (0)	0.989
Peripheral vascular disease, % (*n*)	51.5 (18)	44.0 (25)	0 (0)	0.570
Arterial hypertension, % (*n*)	62.9 (22)	76.0 (19)	100 (1)	0.281
Diabetes, % (*n*)	31.5 (11)	20.0 (5)	0 (0)	0.323
Cancer, % (*n)*	42.9 (15)	8.0 (2)	0 (0)	**0.03**
Chronic kidney disease, % (*n*)	25.7 (9)	32.0 (8)	100 (1)	0.594
Asthma, % (*n*)	2.9 (1)	4.0 (1)	100 (1)	0.808
Chronic obstructive pulmonary disease, % (*n*)	20.0 (7)	4.0 (1)	0 (0)	0.07
Immunodeficiency, % (*n*)	28.6 (10)	0.0 (0)	0 (0)	**0.003**
SpO_2_ < 90%, % (*n*)	56.7 (17)	70.0 (14)	100 (1)	0.341
Pneumonia onset, % (*n*)	80.0 (28)	84.0 (21)	100 (1)	0.693
Need for antibiotics	94.5 (33)	96.0 (24)	100 (1)	0.763

*—defined as the co-occurrence of two or more chronic conditions; **—RSV was omitted from the comparison due to only one fatal case. *p*-values below 0.05 were bolded.

## Data Availability

The data that support the findings of this study are available from the corresponding author upon reasonable request.

## References

[1] Zhang Z.-Q., Li J.-Y., Wang H., Fu C.-Y., Li Y.-L., Guo Q., Bao Y.-W., Wu J., Liao J.-C., Song Y.-Q. (2025). Global, Regional and National Burden of Respiratory Infections among Children and Adolescents under 19 Years of Age from 1990 to 2021 and Projected Trends to 2040. Egypt. J. Bronchol..

[2] (2025). GBD 2021 Upper Respiratory Infections Otitis Media Collaborators Global, Regional, and National Burden of Upper Respiratory Infections and Otitis Media, 1990–2021: A Systematic Analysis from the Global Burden of Disease Study 2021. Lancet Infect. Dis..

[3] Hanage W.P., Schaffner W. (2025). Burden of Acute Respiratory Infections Caused by Influenza Virus, Respiratory Syncytial Virus, and SARS-CoV-2 with Consideration of Older Adults: A Narrative Review. Infect. Dis. Ther..

[4] Maleki F., Welch V., Lopez S.M.C., Cane A., Langer J., Enstone A., Markus K., Wright O., Hewitt N., Whittle I. (2023). Understanding the Global Burden of Influenza in Adults Aged 18–64 Years: A Systematic Literature Review from 2012 to 2022. Adv. Ther..

[5] Scott A., Ansari W., Khan F., Chambers R., Benigno M., Di Fusco M., McGrath L., Malhotra D., Draica F., Nguyen J. (2024). Substantial Health and Economic Burden of COVID-19 during the Year after Acute Illness among US Adults at High Risk of Severe COVID-19. BMC Med..

[6] Rzymski P., Poniedziałek B., Zarębska-Michaluk D., Tomasiewicz K., Flisiak R. (2025). High Seroprevalence and High Risk: Why Are Older Adults More Prone to Respiratory Syncytial Virus?. J. Virol..

[7] Rousogianni E., Perlepe G., Boutlas S., Rapti G., Gouta E., Mpaltopoulou E., Mpaltopoulos G., Papagiannis D., Exadaktylos A., Gourgoulianis K.I. (2025). Clinical Features and Outcomes of Viral Respiratory Infections in Adults during the 2023–2024 Winter Season. Sci. Rep..

[8] Branche A., Ramesh M., Francis B. (2025). A Narrative Review of Key Risk Factors for Severe Illness Following SARS-CoV-2, Influenza Virus, and Respiratory Syncytial Virus Infection. Infect. Dis. Ther..

[9] Choi Y.J., Song J.Y., Wie S.-H., Choi W.S., Lee J., Lee J.-S., Kim Y.K., Kim S.W., Lee S.H., Park K.-H. (2025). Severity of Respiratory Viral Diseases and the Impacts of Underlying Medical Conditions during the Omicron Subvariant Dominant Epidemics-A Comparative Study of SARS-CoV-2, Influenza Virus and Respiratory Syncytial Virus. Pathogens.

[10] Wiemken T.L., Khan F., Puzniak L., Yang W., Simmering J., Polgreen P., Nguyen J.L., Jodar L., McLaughlin J.M. (2023). Seasonal Trends in COVID-19 Cases, Hospitalizations, and Mortality in the United States and Europe. Sci. Rep..

[11] Meslé M.M.I., Sinnathamby M., Mook P., Pebody R., Lakhani A., Zambon M., Popovici O., Lazăr M., Ljubović A.D., WHO European Region Respiratory Network Group (2023). Seasonal and Inter-Seasonal RSV Activity in the European Region during the COVID-19 Pandemic from Autumn 2020 to Summer 2022. Influenza Other Respi. Viruses.

[12] Mboumba Bouassa R.-S., Lukumbisa S., Bélec L. (2025). Simultaneous Detection and Differentiation of SARS-CoV-2, Influenza A/B, and Respiratory Syncytial Viruses in Respiratory Specimens Using the VitaSIRO Solo^TM^ SARS-CoV-2/Flu/RSV Assay. Diagnostics.

[13] Ledda C., Maltezou H.C., Isola G., Motta G., Anfuso C.D., Costantino C., Rapisarda V. (2025). Decoding Point-of-Care Testing: A Strategic Tool for Influenza and SARS-CoV-2 Management in Healthcare Facilities. Biosens. Bioelectron. X.

[14] Meyer J., Gosert R., Bingisser R., Nickel C.H., Tschudin-Sutter S., Leuzinger K. (2025). Diagnostic Performance of a Combined Rapid Antigen Test for Detecting SARS-CoV-2, Influenza Virus, and Respiratory Syncytial Virus in Symptomatic Patients in Tertiary Care. J. Med. Virol..

[15] Kim J.-A., Kang M., Yu H.-J., Kim T.Y., Huh H.J. (2026). Evaluation of the QuickFinder COVID-19/Flu/RSV Antigen Test for Rapid Simultaneous Detection of SARS-CoV-2, Influenza A/B Viruses, and Respiratory Syncytial Virus. Diagn. Microbiol. Infect. Dis..

[16] Scott J., Abers M.S., Marwah H.K., McCann N.C., Meyerowitz E.A., Richterman A., Fleming D.F., Holmes E.J., Moat L.E., Redepenning S.G. (2025). Updated Evidence for Covid-19, RSV, and Influenza Vaccines for 2025-2026. N. Engl. J. Med..

[17] Dewey G., Meyer A.G., Garcia R.G., Santillana M. (2026). Uncovering the Post-Pandemic Timing of Influenza, RSV, and COVID-19 Driving Seasonal Influenza-like Illness in the United States: A Retrospective Ecological Study. Lancet Reg. Health Am..

[18] Hodinka R.L. (2016). Respiratory RNA Viruses. Microbiol. Spectr..

[19] Rasmussen S.A., Jernigan D.B. (2026). Antigenic Drift and Antivaccine Shift in the 2025–2026 Influenza Season. N. Engl. J. Med..

[20] Raharinirina N.A., Gubela N., Börnigen D., Smith M.R., Oh D.-Y., Budt M., Mache C., Schillings C., Fuchs S., Dürrwald R. (2025). SARS-CoV-2 Evolution on a Dynamic Immune Landscape. Nature.

[21] Piñana M., González-Sánchez A., Andrés C., Vila J., Creus-Costa A., Prats-Méndez I., Arnedo-Muñoz M., Saubi N., Esperalba J., Rando A. (2024). Genomic Evolution of Human Respiratory Syncytial Virus during a Decade (2013–2023): Bridging the Path to Monoclonal Antibody Surveillance. J. Infect..

[22] Rzymski P., Poniedziałek B., Zarębska-Michaluk D., Brydak L., Jackowska T., Antczak A., Tomasiewicz K., Flisiak R. (2026). Delineating RSV-A and RSV-B: Epidemiological Patterns, Diagnostics, Clinical Severity, and Preventive Approaches. J. Med. Virol..

[23] Flisiak R., Zarębska-Michaluk D., Brzdęk M., Rorat M., Dobrowolska K., Kozielewicz D., Stankiewicz M., Moniuszko-Malinowska A., Rogalska M., Supronowicz Ł. (2025). Differences in the Clinical Course of COVID-19 in Patients Hospitalized in the 2023/2024 and 2024/2025 Seasons. J. Clin. Med..

[24] Rzymski P., Pleśniak R., Piekarska A., Sznajder D., Moniuszko-Malinowska A., Tomasiewicz K., Skwara P., Zarębska-Michaluk D., Turzańska K., Piasecki M. (2025). Tracking Clinical Severity of Influenza in Adult Hospitalized Patients in 2024: Data from the FluTer Registry in Poland. Vaccine.

[25] Ozeki S., Oshiro M., Fukumi D., Takeuchi T., Mii S., Nishikado Y. (2022). Change over Time in Seasonality and Severity of Children Hospitalized with Respiratory Syncytial Virus Infection in Japan. Pediatr. Infect. Dis. J..

[26] Flisiak R., Jaroszewicz J., Kozielewicz D., Kuchar E., Parczewski M., Pawłowska M., Piekarska A., Rzymski P., Simon K., Tomasiewicz K. (2025). Management of SARS-CoV-2 Infection-Clinical Practice Guidelines of the Polish Association of Epidemiologists and Infectiologists, for 2025. J. Clin. Med..

[27] World Health Organization Clinical Practice Guidelines for Influenza. https://www.who.int/publications/i/item/9789240097759.

[28] Antczak A., Barczyk A., Biesiada A., Czajkowska-Malinowska M., Flisiak R., Gil R., Grabowski M., Kania A., Konstanty M., Krenke R. (2025). RSV Prevention in Adults: Consensus of Experts of Polish Scientific Societies. Fam. Med. Prim. Care Rev..

[29] Wrześniewska-Wal I., Sękowski K., Silczuk A., Jankowski M., Waszyk-Nowaczyk M., Grudziąż-Sękowska J. (2026). Social Attitudes towards Public Health Interventions in Community Pharmacies in Poland: A Nationwide Cross-Sectional Survey. Med. Sci. Monit..

[30] Survey Report on National Seasonal Influenza Vaccination Recommendations and Coverage Rates in EU/EEA Countries, 2024/25. https://www.ecdc.europa.eu/en/publications-data/survey-report-national-seasonal-influenza-vaccination-recommendations-and.

[31] Raport o Chorobach Zakaźnych—Ezdrowie.gov.pl. https://ezdrowie.gov.pl/portal/home/badania-i-dane/raport-o-chorobach-zakaznych.

[32] Rao S., Armistead I., Tyler A., Lensing M., Dominguez S.R., Alden N.B. (2023). Respiratory Syncytial Virus, Influenza, and Coronavirus Disease 2019 Hospitalizations in Children in Colorado during the 2021-2022 Respiratory Virus Season. J. Pediatr..

[33] Balas W.M., Śliwczyński A., Olszewski P., Gołębiak I., Sybilski A.J. (2023). Comparative Analysis of Symptomatology in Hospitalized Children with RSV, COVID-19, and Influenza Infections. Med. Sci. Monit..

[34] Xu S.-W., Ilyas I., Weng J.-P. (2023). Endothelial Dysfunction in COVID-19: An Overview of Evidence, Biomarkers, Mechanisms and Potential Therapies. Acta Pharmacol. Sin..

[35] Levinson T., Wasserman A., Shenhar-Tsarfaty S., Halutz O., Shapira I., Zeltser D., Rogowski O., Berliner S., Ziv-Baran T. (2023). Comparative Analysis of CRP as a Biomarker of the Inflammatory Response Intensity among Common Viral Infections Affecting the Lungs: COVID-19 versus Influenza A, Influenza B and Respiratory Syncytial Virus. Clin. Exp. Med..

[36] Surie D., Yuengling K.A., DeCuir J., Zhu Y., Lauring A.S., Gaglani M., Ghamande S., Peltan I.D., Brown S.M., Ginde A.A. (2024). Severity of Respiratory Syncytial Virus vs COVID-19 and Influenza among Hospitalized US Adults. JAMA Netw. Open.

[37] Xing Y., Bahl A. (2025). Comparative Analysis of Severe Clinical Outcomes in Hospitalized Patients with RSV, Influenza, and COVID-19 across Early and Late COVID-19 Pandemic Phases (2021–2024). J. Clin. Med..

[38] Wee L.E., Lim J.T., Ho R.W.L., Chiew C.J., Young B., Venkatachalam I., Sim J.X.Y., Cheong H.Y., Ng T.Y., Yung C.-F. (2025). Severity of Respiratory Syncytial Virus versus SARS-CoV-2 Omicron and Influenza Infection amongst Hospitalized Singaporean Adults: A National Cohort Study. Lancet Reg. Health West. Pac..

[39] Rzymski P., Sznajder D., Wajdowicz M., Moniuszko-Malinowska A., Pazgan-Simon M., Skwara P., Hlebowicz J., Sikorska K., Piasecki M., Flisiak R. (2026). COVID-19 versus Influenza Hospitalizations in Late 2025 in Poland: A Multicenter Retrospective Comparison. Vaccines.

[40] Rzymski P., Piekarska A., Pleśniak R., Sznajder D., Zarębska-Michaluk D., Tomasiewicz K., Piasecki M., Pazgan-Simon M., Hlebowicz J., Turzańska K. (2025). Unraveling Poland’s Unprecedented Influenza Surge in Early 2025: Increased Viral Severity or Post-Pandemic Vulnerability?. Pharmacol. Rep..

[41] Tsioutis C., Walkowiak M.P. (2026). Post-Pandemic Challenges: Endemic COVID-19, Vaccine Hesitancy, and Viral Resurgence. Trop. Med. Infect. Dis..

[42] Hotez P. (2025). It Won’t End with COVID: Countering the next Phase of American Antivaccine Activism 2025-29. PLoS Glob. Public Health.

[43] Jeannoël M., Lina G., Rasigade J.P., Lina B., Morfin F., Casalegno J.S. (2019). Microorganisms Associated with Respiratory Syncytial Virus Pneumonia in the Adult Population. Eur. J. Clin. Microbiol. Infect. Dis..

[44] Bartley P.S., Deshpande A., Yu P.-C., Klompas M., Haessler S.D., Imrey P.B., Zilberberg M.D., Rothberg M.B. (2022). Bacterial Coinfection in Influenza Pneumonia: Rates, Pathogens, and Outcomes. Infect. Control Hosp. Epidemiol..

[45] Fan H., Zhou L., Lv J., Yang S., Chen G., Liu X., Han C., Tan X., Qian S., Wu Z. (2023). Bacterial Coinfections Contribute to Severe COVID-19 in Winter. Cell Res..

[46] Havers F.P., Hicks L.A., Chung J.R., Gaglani M., Murthy K., Zimmerman R.K., Jackson L.A., Petrie J.G., McLean H.Q., Nowalk M.P. (2018). Outpatient Antibiotic Prescribing for Acute Respiratory Infections during Influenza Seasons. JAMA Netw. Open.

[47] Miller L., Beaney T., Hope R., Cunningham M., Robotham J.V., Pouwels K.B., Costelloe C.E. (2025). General Practice Antibiotic Prescriptions Attributable to Respiratory Syncytial Virus by Age and Antibiotic Class: An Ecological Analysis of the English Population. J. Antimicrob. Chemother..

[48] (2024). GBD 2021 Antimicrobial Resistance Collaborators Global Burden of Bacterial Antimicrobial Resistance 1990-2021: A Systematic Analysis with Forecasts to 2050. Lancet.

[49] Rzymski P. (2026). Potential Non-Specific Benefits of Seasonal Influenza Vaccination: Evidence, Knowledge Gaps, and Future Directions. Vaccines.

[50] Barchitta M., Maugeri A., Vinci R., Agodi A. (2022). The Inverse Relationship between Influenza Vaccination and Antimicrobial Resistance: An Ecological Analysis of Italian Data. Vaccines.

[51] Chartrand C., Tremblay N., Renaud C., Papenburg J. (2015). Diagnostic Accuracy of Rapid Antigen Detection Tests for Respiratory Syncytial Virus Infection: Systematic Review and Meta-Analysis. J. Clin. Microbiol..

[52] Łuniewska K., Rzymski P., Poniedziałek B., Szymański K., Kondratiuk K., Czajkowska E., Mańkowski B., Brydak L.B. (2026). Monitoring of RSV-A and RSV-B Circulation in Poland across Three Post-Pandemic Seasons (2022–2025). Viruses.

[53] Sharma S.P., Rathore B.K., Meena M., Sharma N. (2025). Clinical Severity and Hospitalization Burden of Influenza Subtypes: A Cross-Sectional Analysis from a Tertiary Care Center in North India. Cureus.

